# Lower Mortality Associated With Overweight in the U.S. National Health Interview Survey: Is Overweight Protective?

**DOI:** 10.1097/MD.0000000000002424

**Published:** 2016-01-15

**Authors:** Zhiqiang Wang, Meina Liu, Tania Pan, Shilu Tong

**Affiliations:** From the University of Queensland, School of Medicine, Herston, Queensland, Australia (ZW, TP); Department of Biostatistics, Public Health College, Harbin Medical University, Harbin, China (ML); and School of Public Health and Social Work, Institute of Health and Biomedical Innovation, Queensland University of Technology, Brisbane, Queensland, Australia (ST).

## Abstract

It is still debatable whether overweight has protective or detrimental effects on survival. The focus of the ongoing debate is on possible confounding bias due to factors such as preexisting illness and smoking. We aimed to assess the association between overweight and mortality and to examine confounding effects of various factors including smoking and preexisting cancer, cardiovascular disease (CVD), diabetes, asthma, bronchitis, and kidney disease on the overweight–mortality association in adults.

The data were extracted from the public-use National Health Interview Survey (NHIS) 1997 to 2009. Mortality data up to December 31, 2011 were linked to 131,813 with normal weight and 120,217 overweight adults. We assessed the association between overweight and mortality using Cox proportional hazard model with adjustments for various sets of confounding factors—age, sex, smoking, race, survey year, diabetes, CVD, cancer, asthma, bronchitis, and kidney disease.

During the period from the original surveys to December 31, 2011, 22,513 (11,815 normal weight and 10,698 overweight) adults died. Normal weight and overweight groups differed in the characteristics of age, sex, smoking, and preexisting diseases. After adjusting for age and sex, the risk of dying was lower for overweight than normal weight adults (hazard ratio [HR], 0.82; 95% confidence interval [CI]: 0.80–0.85). Lower mortality risk associated with overweight remained after further adjusting for smoking and preexisting diseases such as diabetes, CVD, cancer, asthma, bronchitis, and kidney disease (HR, 0.80; 95% CI: 0.78–0.82). We observed a similar pattern for men and women, and for those free from preexisting diabetes, hypertension, and CVD.

In conclusion, overweight adults have a lower mortality risk than normal weight adults. Our findings do not support that the lower mortality in overweight adults is due to confounding effects of smoking and preexisting diseases.

## INTRODUCTION

Although researchers have consistently shown that underweight and higher levels of obesity are associated with an increased mortality relative to normal weight,^1–3^ it is still debatable whether overweight has protective or detrimental effects on survival. A lower mortality risk among overweight relative to normal weight adults is often reported in previous studies as shown in a systematic review by Flegal et al.^1^ However, it has been argued that reverse causation owing to preexisting chronic illness and inadequate control for smoking status can distort the true association between overweight and mortality, because preexisting illness and smoking are associated with both decreased body mass index (BMI) and increased mortality.^2,4,5^ However, previous studies addressing this issue through adjustments and exclusions some conditions found little or no effect of these factors on the association between overweight and mortality.^1^ Contrary to a protective effect of overweight on survival, several studies have shown no difference in all-cause mortality between overweight and normal weight^6–8^ while others reported a higher mortality risk in overweight than in normal weight adults.^2,9–11^

The focus of the ongoing debate is on possible confounding bias due to factors such as preexisting illness and smoking.^12–14^ Although the confounding bias is a possible explanation of the lower mortality risk associated with overweight, empirical evidence is still lacking to support or oppose this. One challenge of appropriately adjusting for confounding effects is the lack of sufficient data on potential confounding factors with a large sample size. It is also a challenge to identify appropriate statistical models to minimize over-adjustment.^15^ The public-use data from the US National Health Interview Survey (NHIS) provide an opportunity to assess the impact of smoking and preexisting illness on the association between overweight and mortality.

In this study, we compared mortality risks of overweight and normal weight adults in the NHIS (1997–2009) and assessed confounding effects of age, sex, smoking, race, survey year, and preexisting diseases such as diabetes, cardiovascular disease (CVD), cancer, asthma, bronchitis, and kidney disease.

## METHODS

### Participants and Study Groups According to BMI Values

Data on BMI and potential confounding factors extracted from the Sample Adult Data Files of 13 waves of the public-use NHIS data from 1997 to 2009.^16^ Those data were linked to the public-use version of the NHIS Linked Mortality Files^17^ on death records through December 2011. BMI was calculated as weight in kilograms divided by the square of height in meters, derived from information that respondents supplied in response to survey questions regarding self-reported height and weight.

**FIGURE 1:**
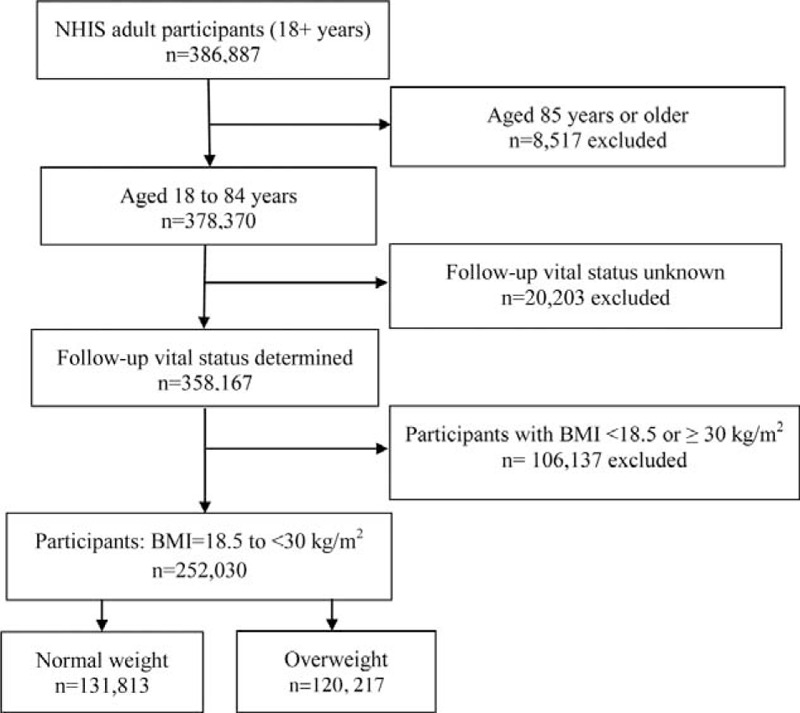
Flow chart of the selection of participants for the present study.

BMI values were categorized into underweight (<18.5), normal weight (18.5–24.9), overweight (25–29.9), and obese (30 + km/g^2^). To focus on the comparison between overweight and normal weight groups, we excluded underweight and obese participants. As shown in Figure 1, there were 386,887 participants in the original survey. We also excluded those 85 years or older and those with follow-up vital status unknown. A total of 252,030 adults aged 18 to 84 years had BMI from 18.5 up to 30 kg/m^2^ and were eligible for mortality linkage. Among them, 131,813 were normal weight while 120,217 were overweight.

### Mortality

The public-use linked mortality records were used to identify those who had died during the period from the original surveys (1997–2009) to December 2011. Eligible individuals who had no death records were considered survived beyond December 31, 2001.

### Potential Confounding Variables

Age, sex, smoking status, survey year, race, and preexisting CVD, diabetes, cancer, asthma, bronchitis, and kidney disease were considered as potential confounding factors. Smoking status was categorized as self-reported nonsmoker, former smoker, and current smoker. Preexisting CVD, diabetes, cancer, asthma, bronchitis, and kidney disease were also determined through self-reporting. A preexisting CVD was defined as being diagnosed with one of the following self-reported conditions: coronary heart disease, angina pectoris, heart attack, heart disease, and stroke. Preexisting cancers included a history of any types of cancers. Participants were also divided into 3 racial groups: white, black, and others.

### Data Analyses

The association between overweight and mortality was assessed using Cox proportional hazard models. The survival time was the time difference between the date of original survey and the date of death for those who died or December 31, 2011 for those survived. Various hazard ratios (HRs) were calculated after adjusting for different sets of confounding factors in a stepwise fashion.^18^ In step 1, the crude HR was calculated without adjusting for any covariates. In step 2, the HR was calculated after adjusting for age and sex only. In step 3, we added each of the remaining variables to the previous Cox proportional model one at a time and compared each HR with the one obtained in the previous step. The computer program^18^ chose the variable that caused the largest change in HR and kept this variable in the model. In the following steps, the program repeated step 3 for the remaining variables and chose one variable at a time until all variables were added into the model. We presented the HRs of mortality for overweight and changes in hazards ratios in all steps to inspect confounding effects from those factors. We also performed analyses for men and women separately. To address the issue of possible over-adjustment (adjusting for variables in the causal pathway),^1^ we analyzed data from those who were free from the following conditions: diabetes, hypertension, and CVD on the original survey, since those conditions were considered intermediates between overweight and mortality. We performed a sensitivity analysis based on the data from never smokers who were free from preexisting hypertension, diabetes, CVD, cancer, bronchitis, kidney disease, cancer or asthma on a smaller sample of 92,003 (37%) participants. In all tests, *P* values were 2-sided, and *P* < 0.05 was considered to be statistically significant. We used the *chest* command in Stata to examine confounding effects^18^ and Stata/SE 13 for all other analyses.^19^ This project was approved by the University of Queensland Behavioural & Social Sciences Ethical Review Committee.

## RESULTS

During the period of 21,287,085 person-years, 22,513 participants died, including 11,815 with normal weight and 10,698 with overweight. Overweight adults were different from normal adults in all characteristics listed in Table 1. They were older and more likely to be men. They had a higher prevalence of diabetes, hypertension, CVD, cancer, bronchitis, and kidney disease. Overweight adults were less likely to be current smokers and more likely to be former smokers. Therefore, it is important to take potential confounding effects of those variables into consideration when assessing the association between overweight and mortality.

**TABLE 1:**
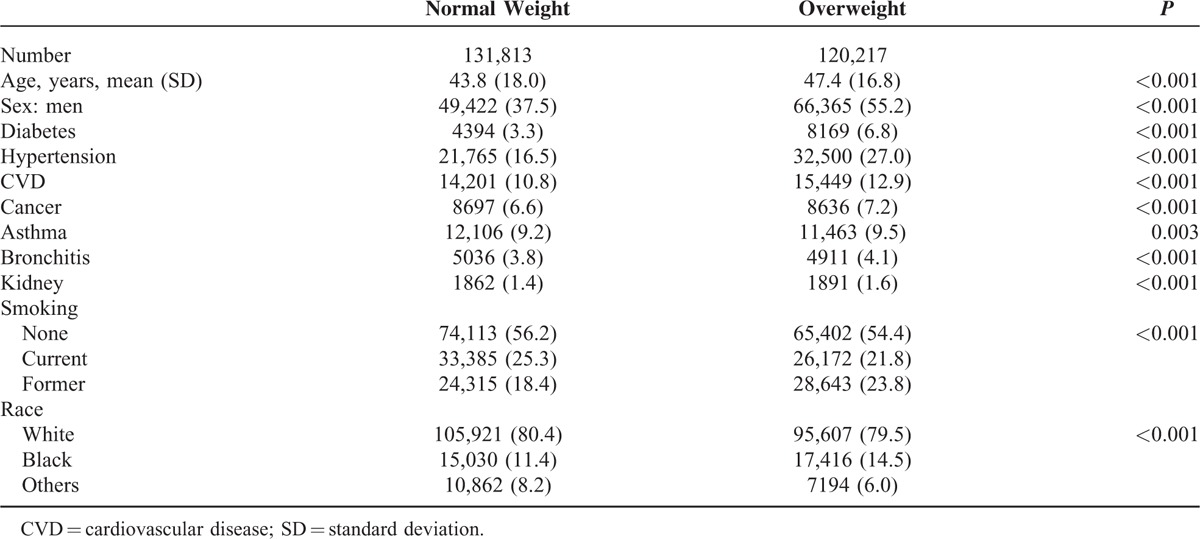
The Characteristics of Normal Weight and Overweight Adults Based on Data From the National Health Interview Survey 1997–2009

**FIGURE 2:**
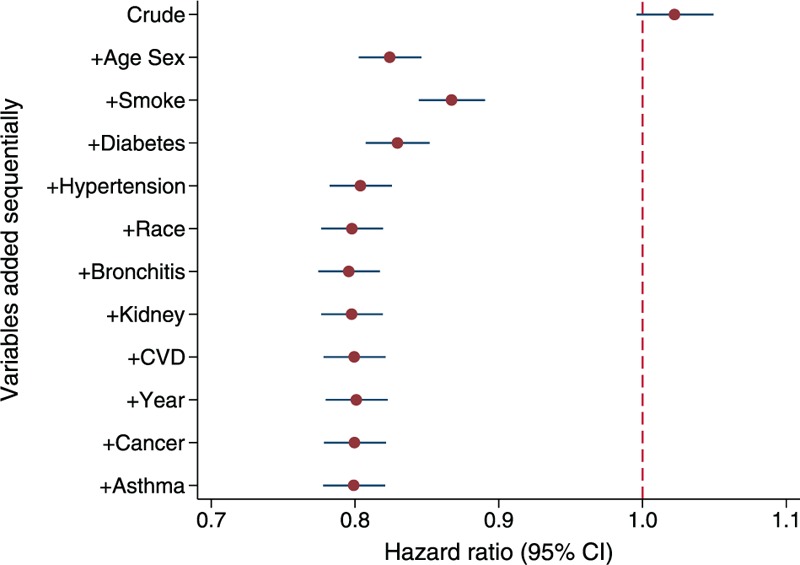
Hazard ratios of mortality in adults: overweight (25 to <30 kg/m^2^) relative to normal weight (BMI 18.5 to <25 kg/m^2^).

Table 2 shows HRs by adding potential confounding factors one at a time according to the changes in HR. The crude HR in step 1 indicates that overweight adults appeared to have a slightly higher (2%) mortality risk than normal weight (HR = 1.02; 95% confidence interval [CI]: 1.00–1.05). However, this HR might not reflect the true association between overweight and mortality due to potential confounding bias as overweight and normal weight groups were different in a number of important factors on the original surveys. In step 2, we calculated the HR after adjusting for age and sex, aiming to compare mortality risks between overweight and normal adults with the same age and sex without considering other factors. Overweight adults had approximately 18% lower risk of dying (HR = 0.82; 95% CI: 0.80–0.85) than normal weight adults of the same age and sex. From steps 3 to 11, at each step, the computer program^18^ automatically chose one variable that had caused the largest change in the HR at a time and added it into the Cox proportional hazard model. The changing value in HR at each step indicated the magnitude of the confounding effect. Those HR s changed moderately by further adjusting for smoking, diabetes, and hypertension, and the mortality risk for the overweight group was about 20% lower than that for the normal weight group (HR = 0.80; CI: 0.78–0.83). Further adjusting for the remaining factors of race, survey year, preexisting bronchitis, kidney disease, CVD, cancer, and asthma had little impact on the strength of the association between overweight and mortality (Figure 2).

**TABLE 2:**
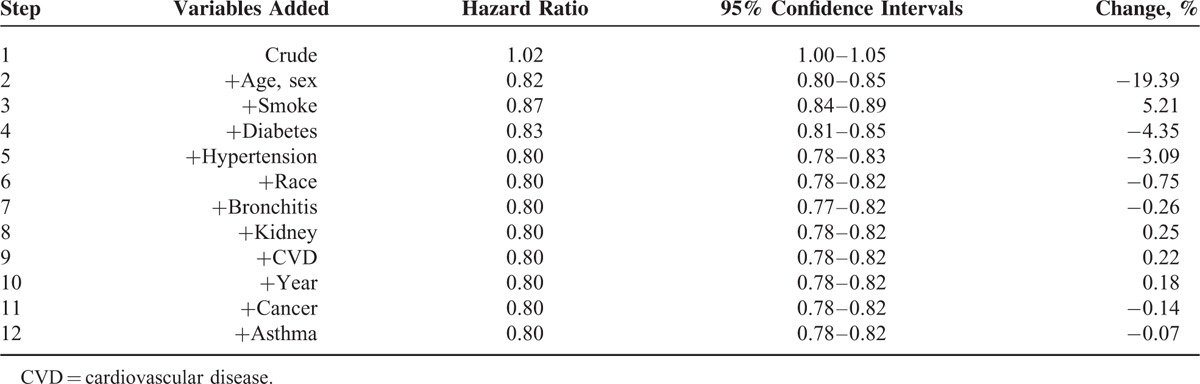
Hazard Ratios and Changes in Hazard Ratios by Adding Potential Confounding Factors in a Stepwise Fashion

**FIGURE 3:**
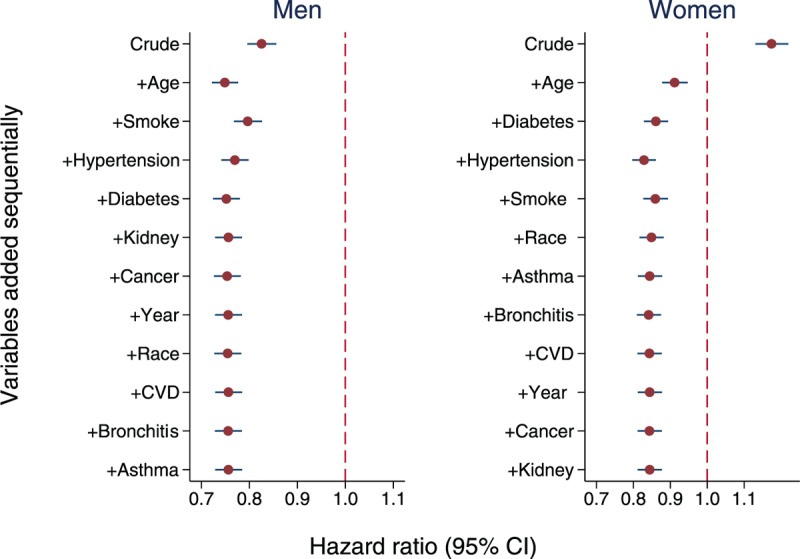
Hazard ratios of mortality in adults: overweight relative to normal weight for men and women.

We performed analyses for men and women separately. The crude HRs showed that overweight increased the mortality risk in women and decreased the risk in men. However, after adjusting for age, overweight was associated with decreased mortality in both genders (Figure 3). In addition to age, the first 3 variables which caused the largest changes in HR estimates in each of the following 3 steps included smoking, hypertension, and diabetes in both genders. Further adjusting for other variables had little impact on HR estimates.

**FIGURE 4:**
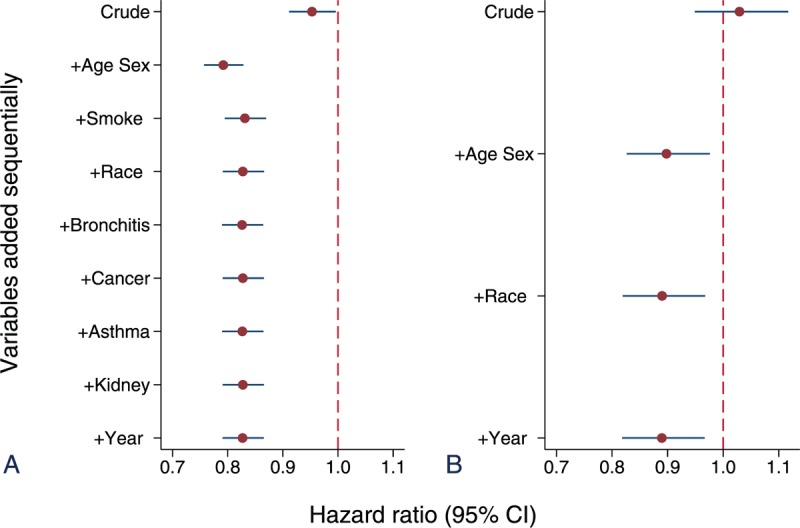
Hazard ratios of mortality: overweight relative to normal weight among adults free from diabetes, hypertension and cardiovascular disease at original survey (A), and among never smokers free from preexisting known diseases (B).

We further excluded those with preexisting hypertension, CVD, and diabetes as those variables were considered intermittent factors in the causal pathway. Among those free from those conditions, overweight was still associated with decreased mortality after adjusting for age, sex, and smoking (HR = 0.83; 95% CI: 0.80–0.87). Further adjusting for other factors had little impact on HR estimates (Figure 4A). Even for never smokers free from preexisting diseases, although the sample size was much smaller and the strength of the association was not as strong as that estimated using the whole sample, overweight was still associated with decreased mortality (HR = 0.89; 95% CI, 0.82–0.97) (Figure 4B).

## DISCUSSION

This study focused on the association between overweight and mortality in adults based on a large population-based dataset from the NHIS. We found that overweight was associated with about 20% decreased mortality risk relative to normal weight. Since overweight and normal weight adults were substantially different in a number of factors including smoking and preexisting diseases on the original surveys, we assessed those factors as potential confounders in this study. Without adjusting for confounding factors, overweight appeared to be associated with increased risk of mortality. However, this observed crude association was likely to be due to confounding effects because after adjusting for age and sex, overweight adults was associated with lower mortality relative to normal weight. Further adjusting for smoking, preexisting diabetes and hypertension only slightly altered the HR estimate. Adjusting for additional variables of preexisting cancer, CVD, bronchitis, kidney disease, and asthma had little impact on the direction and magnitude of the association. Therefore, it is unlikely that the lower mortality risk in overweight adults was attributable to the confounding effects of smoking and preexisting diseases in this study.

A number of studies show overweight as a protective factor^20–22^ while others a risk factor for mortality.^2,9–11^ Adams et al^2^ reported that overweight was associated with 20% to 40% increase in the risk of death among healthy people who had never smoked. On the other hand, Orpana et al^23^ reported that overweight was associated with 17% decreased risk of death in Canadian adults. Flegal et al^3,24^ also reported that overweight was not a risk factor for mortality. In a systematic review, overweight is associated with lower mortality relative to the normal weight group with an overall summary HR 0.94 (95% CI: 0.91–0.96).^1^ Unlike previous studies, we specifically focused on comparing mortality risks between overweight and normal weight groups. We found much stronger protective effects of overweight on mortality than previously reported. After adjusting for smoking and preexisting diseases, overweight adults had about 20% lower mortality risk than normal weight adults. Therefore, our findings do not support the suggestion that smoking and preexisting illness are important causes of bias and this is consistent with a previous systematic review and meta-analysis by Flegal et al.^1^

However, confounding bias may play a role in the inconsistent findings in previous studies. Even using the same dataset in this study, crude HR estimates suggest that overweight appeared to be a risk factor for women but a protective factor for men. With adjustment of age, overweight was consistently associated with decreased mortality for both sexes. We found a stronger association than that reported in a published meta-analysis,^1^ possibly due to mixing some studies reporting unadjusted or inappropriately adjusted HRs in the meta-analysis.

We applied a stepwise approach to observe confounding effects of a range of factors. We found that differences in age and sex between overweight and normal weight groups could seriously bias the overweight–mortality association if they are not adjusted for. This stepwise approach is helpful for understanding confounding effects of multiple factors in observational studies.^18^ This is the first study to examine the confounding effects of a large number of major preexisting diseases in a stepwise fashion. The large dataset from the NHIS also enabled us to perform subgroup and sensitivity analyses that showed a lower risk in overweight group in all analyses. This stepwise approach is also useful for understanding over-adjustment of variables in the causal pathway. Because we found that overweight was significantly associated with lower mortality after only adjusting for age and sex, it is unlikely the observed association was due to over-adjustment. Our subgroup and sensitivity analyses also support that overweight was protective against mortality.

There are several limitations in this study. We used self-reported BMI values which are likely to be lower than measured BMI.^25^ However, the association between overweight and mortality are similar for self-reported and measured BMI values.^1^ In this observational study, we could not control the unidentified preexisting illness as in randomized interventional studies. If unidentified illness occurred more frequently in normal weight than in overweight adults in this study, we might overestimate the protective effect of overweight. As shown in Table 1, the identified preexisting illness occurred more frequently in the overweight group than the normal weight group. Therefore, it is unlikely that unidentified preexisting illness would be higher in the normal weight group. The data for this study were derived from the nationally representative survey (NHIS) of the noninstitutionalized adults in the United States. Whether our results are generalizable to other populations remains to be confirmed.

## CONCLUSIONS

Relative to normal weight, overweight is associated with lower all-cause mortality. This association is unlikely to be due to the confounding effects of smoking and preexisting illness or due to the over-adjustment of intermittent variables in the causal pathway. Our findings support the notion that overweight is protective against mortality in adults.
